# The Effect of *Aloe Vera* Gel and Nitrofurazone on Dressing Related Pain of Superficial Burn Wounds

**Published:** 2017-05

**Authors:** Shokoh Varaei, Fatemeh Mohaddes Ardabili, Parichehr Sabaghzadeh Irani, Hadi Ranjbar

**Affiliations:** 1 School of Nursing and Midwifery, Tehran University of Medical Sciences, Tehran, Iran;; 2 School of Nursing and Midwifery, Iran university of Medical Sciences, Tehran, Iran

**Keywords:** Aloe vera, Nitrofurazone, Pain, Burn, Wound

**DEAR EDITOR**

Burn injuries are a painful form of trauma.^1^ Patients of burn injuries experience severe pain on a daily basis, both immediately after the injury and during therapeutic procedures, such as dressing changes,debridement and physiotherapy.^2^ In addition, repetition of these painful procedures often creates anticipatory anxiety forpatients with burns.^3^ Anxiety induced by a bad acute pain experience risks poor compliance with rehabilitation therapies,increased pain perception and loss of belief in the burn team.^3^ Application of topical anti-bacterial agents and disinfectants was shown as the most widely used topical therapy in burn injuries with anti-microbial effects.^4^ Herbal medicines with less toxicity and as inexpensive therapies have been used in healing of burn injuries,^5^^,^^6^ but reports on pain control in burn patients is very few.

Aloe vera (family: Liliaceae) has been used in traditional medicine for a long time. It is one of the most recognizableherbs in the world and the medicinal part is the succulentleaves. A topical skin gel provides wonderful healingsupport for the skin. Aloe vera contains many importantnutrients for the body, including amino acids, B vitamins,and other nutrients that support general health. It also haspharmacological properties including antioxidant, wound healing, antibacterial, antifungal, and immunomodulatingeffects.^7^

Thirty patients were selected non-randomly among the outpatients referring to Burn Section of Kerman Shafa Hospital, Kerman, Iran; but symmetric organs were selected with random assignment method. The inclusion criteria included outpatients with the second degree burn below 20% without infection in two symmetric organs which should be dressed with Nitrofurazone ointment, wound should not be contaminated with the contaminants and they should not be affiliated with metabolic diseases such as cancer, AIDS, allergy and dermal diseases. They should tend to participate in the research and be able to respond to the questions. Informed consent letters were provided from all patients. 

Each patient randomly selected a treatment. It is necessary to note that these areas were similar in two sides of the body and had equal burn degree. Wounds were washed every day with normal 0.9% saline and 2% nitrofurazone ointment. In the intervention area, *Aloe vera* gel was used after washing the wound with normal saline. Dressings were changed every day. The burn areas were assessed in terms of daily infection and the wound was regarded infectious in case of each of the symptoms, swelling in the burnt area, change in color of the burn wound to dark red, purulent, odorous secretions and fever during treatment and the sample was excluded from the study and another sample replaced it. 

The treatment method changed according to view of the physician. *Aloe vera* gel which was prepared as 100% mucilage and sterile gel from the middle part of the leaf of *Aloe vera* gel in the Herbs Research Center of Karaj Jihad University. In a questionnaire, demographic specifications included individual age, gender, marital status, etc. were recorded through interview. To determine local pain of the burn wound, pain intensity was assessed 10 min before change of dressing and 24, 48 and 72 hours after dressing with 2% nitrofurazone ointment and *Aloe vera* gel using visual analogue scale. 

 Most of the studied samples were in age group of 15-30 years. In terms of gender, 46.7% of the patients were men and 53.3% of the patients were women. The maximum BMI of the participants in the research was 26-30. The maximum percent of pain intensity had been expressed in the intervention area (63.3%) and intensive pain had been expressed in the control area (60%), 10 min before start of intervention; but there was no statistically significant difference between two areas, therefore, two areas were homogenous in terms of pain intensity. 

The maximum pain intensity reported in control area was severe pain and the medium pain was reported in the intervention area, 24 hours after start of intervention. The maximum pain intensity was medium pain without expression of weak pain in the control area, 48 hours after start of intervention; while medium pain along with weak pain was reported in the intervention area. The maximum pain intensity was medium pain in the control area and weak pain was reported in the intervention area, 72 hours after start of intervention. There was statistically significant difference in the control area and intervention area in terms of pain intensity 24, 48 and 72 hours after start of intervention (*p*=0.0001, *p*=0.002, *p*=0.0001). 

So dressing pain intensity decreased significantly during a 72-hour period in both areas (*p*=0.001), but *Aloe vera* gel could reduce pain more and faster than nitrofurazone. Perhaps, the reason may be the presence of carboxy peptidase in *Aloe vera* which inactivates bradykinin which is the powerful factor of acute inflammatory pain.^8^ Magnesium lactate in *Aloe vera* gel is used as anti-itching and analgesic drug by inhibiting histidine-decarboxylase which controls conversion of histidine to histamine in mast cells.^9^

It is necessary to note that although the patients did not know type of dressing at time of dressing, they were satisfied with ease of dressing and painless spot of the burnt wound at time of dressing with *Aloe vera* gel and the reason was ease and painlessness of wound after dressing which can be a reason for alleviation of pain at time of dressing with *Aloe vera* gel in patients with second degree burn. 

## References

[1] Manafi A, Kohanteb J, Mehrabani D, Japoni A, Amini M, Naghmachi M, Zaghi AH, Khalili N (2009). Active immunization using exotoxin a confers protection against Pseudomonas aeruginosa infection in a mouse burn model. BMC J Microbiol.

[2] Tanideh N, Rokhsari P, Mehrabani D, Mohammadi Samani S, Sabet Sarvestani F, Ashraf MJ, Koohi Hosseinabadi O, Shamsian Sh, Ahmadi N (2014). The healing effect of licorice on pseudomonas aeruginosa infected burn wounds in experimental rat model. World J Plast Surg.

[3] Mohades Ardabili F, Purhajari S, Najafi T, Haghani H (2014). The Effect of Shiatsu Massage on Pain Reduction in Burn Patients. World J Plast Surg.

[4] Hazrati M, Mehrabani D, Japoni A, Montasery H, Azarpira N, Hamidian-shirazi AR, Tanideh N (2010). Effect of honey on healing of Pseudomonas aeruginosa infected burn wounds in rat. J Appl Anim Res.

[5] Tanideh N, Haddadi MH, Rokni-Hosseini MH, Hossienzadeh M, Mehrabani D, Sayehmiri K, Koohi-Hossienabadi O (2015). The Healing effect of Scrophularia striata on experimental burn wounds infected to Pseudomonas aeruginosa in rat. World J Plast Surg.

[6] Mehrabani D, Farjam M, Geramizadeh B, Tanideh N, Amini M, Panjehshahin MR (2015). The healing effect of curcumin on burn wounds in rat. World J Plast Surg.

[7] Akhoondinasab MR, Akhoondinasab M, Saberi M (2014). Comparison of healing effect of Aloe vera extract and silver sulfadiazine in burn injuries in experimental rat model. World J Plast Surg.

[8] Yagi A, kabash A, Mizuno K, Moutafa SM, Khalifa TI, Tusuji H (2003). Radical scavenging glycoprotein cyclooxygenase-2 and thromboxane A2 synthase from aloe vera gel. Planta Med J.

[9] Maenthaisong R, Chaiyakunapruk N, Niruntraporn S, Kongkaew C (2007). The efficacy of aloe vera used for burn wound healing. A systematic review. Burns.

